# Automatic Shrimp Fry Counting Method Using Multi-Scale Attention Fusion

**DOI:** 10.3390/s24092916

**Published:** 2024-05-02

**Authors:** Xiaohong Peng, Tianyu Zhou, Ying Zhang, Xiaopeng Zhao

**Affiliations:** 1 Faculty of Mathematics and Computer Science, Guangdong Ocean University, Zhanjiang 524088, China; pngxh@gdou.edu.cn (X.P.); 2112209005@stu.gdou.edu.cn (T.Z.); 2112109009@stu.gdou.edu.cn (X.Z.); 2 Southern Marine Science and Engineering Guangdong Laboratory, Zhanjiang Bay Laboratory, Zhanjiang 524000, China

**Keywords:** smart aquaculture, deep learning, shrimp fry counting, SFCNet, multi-scale attention fusion

## Abstract

Shrimp fry counting is an important task for biomass estimation in aquaculture. Accurate counting of the number of shrimp fry in tanks can not only assess the production of mature shrimp but also assess the density of shrimp fry in the tanks, which is very helpful for the subsequent growth status, transportation management, and yield assessment. However, traditional manual counting methods are often inefficient and prone to counting errors; a more efficient and accurate method for shrimp fry counting is urgently needed. In this paper, we first collected and labeled the images of shrimp fry in breeding tanks according to the constructed experimental environment and generated corresponding density maps using the Gaussian kernel function. Then, we proposed a multi-scale attention fusion-based shrimp fry counting network called the SFCNet. Experiments showed that our proposed SFCNet model reached the optimal performance in terms of shrimp fry counting compared to CNN-based baseline counting models, with MAEs and RMSEs of 3.96 and 4.682, respectively. This approach was able to effectively calculate the number of shrimp fry and provided a better solution for accurately calculating the number of shrimp fry.

## 1. Introduction

Object counting refers to the estimation of the number of objects in a region of interest to accurately obtain information on the number of objects in the area and provide guidance for subsequent related decisions [1,2]. It has been applied in the fields of crowd counting [3], plant counting [4], and vehicle counting [5]. Shrimp fry counting is a basic operation for biomass estimation in aquaculture. The accurate counting of shrimp fry not only serves as a means of assessing the production and reproductive capacity of mature shrimp but also evaluates the survival rate of the shrimp fry in each tank and the control of breeding density and provides instructions for the management of transportation and sales [6]. At present, most shrimp fry counting is performed manually, which is time-consuming and laborious, and the calculation accuracy is low. Meanwhile, it is easy to hurt them and affect the normal growth of the shrimp fry. Therefore, a shrimp fry counting method that can be automated and has high accuracy and efficiency is needed.

With the rapid development of artificial intelligence technology, the emerging field of smart aquaculture has emerged, which aims to improve the yield and efficiency of aquaculture through computer vision and deep learning [7,8]. Shrimp fry counting, as a research direction of smart aquaculture [9], is widely favored by researchers and producers for its high efficiency, low cost, and easy operation. With the aid of a terminal device (e.g., a mobile phone) embedded with this method, fishermen do not need to know the specific details of the method; they only need to take an image of the shrimp fry to automatically obtain the number of shrimp fry. At the same time, our model can also provide more accurate counting results for factory farming.

The existing methods for shrimp fry counting can be divided into two main types: detection-based methods and regression-based methods. Detection-based shrimp fry counting has benefited from strong development in the field of object detection. Zhang [10] used a lightweighted model (LIGHT-YOLOv4) to reduce the complexity of the model. In their experiment, the backbone of YOLOv4 was replaced with the backbone of MobileNetV3 [11]. Although the accuracy was reduced by 2%, the size of the model was reduced to one-sixth of that of the original YOLOv4 model, which can be effectively applied to terminal devices. Feng [12] attempted to solve the problems of overlapping, as well as sticking fish fry in water, and proposed a lightweight object detection counting method (YOLOv4-Tiny) based on deep learning and added an attention mechanism (CBAM), which could satisfy edge computing devices to perform automatic counting while obtaining high counting accuracy. Zhang [13] proposed a dynamic fish fry counting method to compensate for the shortcomings of the current methods, which are all implemented in static scenarios. They regarded fish fry counting as a multi-object tracking problem based on tracking by detection, combined YOLOv5 with SORT, and improved the SORT algorithm based on multi-matching and trajectory recovery, for which the final tracking accuracy reached 82.6%. The recently proposed YOLOv7 [14] and YOLOv8 [15] have a high accuracy and running speed in object detection, which also provides a reliable solution for object counting. However, for small objects such as shrimp fry, due to the small pixels they occupy in the image, they will inevitably lead to missed detection, resulting in counting errors. While regression-based shrimp fry counting methods use a density map as a training label for counting, this method integrates the final predicted density map matrix to obtain the final number of objects, which can better predict the number of objects in the image. Hu [16] proposed a counting model for shrimp larvae that draws on the method of density map estimation used in crowd counting and added a multi-scale module. The results showed that the accuracy of counting more than 1000 shrimp fawns reached 98.72%. Zhang [17] used a generative adversarial network (CycleGAN) to synthesize the dataset, set in a way that avoids heavy manual labeling, and proposed a shrimp egg counting network (SECNet) for implementing the counting process, with a final accuracy of 99.2%. Li [18] proposed a counting method (MSENet) for portable counting devices for fish fry counting. Based on this method, the counting datasets NCAUF and NCAUF-ex were constructed to verify the generalization performance of the network, and the final MAE of the model reached 3.33. Hou [19] improved the multicolumn convolutional neural network (MCNN) for residual bait counting, and experiments showed that the improved MCNN was able to calculate the amount of residual bait efficiently. Liu [20] proposed ShrimpSeed_Net for shrimp seed counting, which was based on the improved CSRNet and was successfully implemented in smartphones with an accuracy of 95.53%.

With the research deepening, many emerging structures can bring significant improvements in model performance. Multi-scale structures can integrate feature maps at different scales so that the network can learn global features and improve the ability to learn local information. In smart aquaculture, past studies have also incorporated multi-scale structures into their models to improve their performance. Zhang [21] analyzed fish feeding behavior. He used MobileNetV3 as the backbone and improved the channel attention module based on multi-scale information fusion. They fused the multi-scale feature map with the original image through the operation of down-sampling, which effectively enhanced the attention to small targets and obtained high-feeding intensity classification accuracy. Yu [22] designed a multi-scale attention mechanism to improve the accuracy of fish counting by designing convolutional layers with different convolutional kernel sizes and obtaining receptive fields at different scales in parallel. Wang [23] used U-Net [24] as the backbone to construct the Multi-scale with Dilated convolution and Offset Attention U-Net (MDOAU-Net), which used multi-scale feature fusion blocks to extract the features of the original input; their method effectively promoted the fusion of different feature maps. The experimental results demonstrated their superior performance compared to seven existing methods. In addition, the attention mechanism allowed the model to focus on the important parts of the image. Li [25] designed a Synergistical Attention Module (SAM), which allowed channel affinity extraction while preserving spatial details, and embedded the module into a Synergistical Attention Perception Network (SAPNet) for the semantic segmentation of remote sensing images, so that the network enriched the inference clues through the required spatial and channel details. The experiment verified the efficiency of the SAM. In order to solve the problem of fish counting in high-density scenarios, Chen [26] added an attention network to the model, which included a nonlinear batch-normalized residual block, a convolutional layer, and two parallel independent convolutional layers. Yu [27] proposed a deep learning network model based on a multi-module and attention mechanism (MAN) to determine farmed fish counts. It included a feature extraction module, an attention module, and a density estimation module. The experiments showed that the method based on an MAN could promote the exploration of correlations in dense fish counting.

In this paper, a shrimp fry counting model based on a fully convolutional neural network (SFCNet) is proposed. This model adopts a regression-based method to achieve shrimp fry counting, which can accurately count shrimp fry in breeding tanks; our counting performance is the best compared with the four other traditional CNN counting networks. The main contributions of this paper are as follows:

- The shrimp fry dataset was collected and labeled. It contained 556 images, of which 390 were used as the training set, 63 as the validation set, and 103 as the test set. The resolution size of the images was 768 × 576;
- A shrimp fry counting network based on multi-scale attention fusion (SFCNet) is proposed, which uses VGG-16 as the frontend to accept images and uses a multi-scale structure and attention mechanism in the backend to improve the global modeling and local information extraction ability of the model. Finally, it outputs a density map with the same size as the original image;
- Our SFCNet achieved an optimal performance (MAE: 3.96, RMSE: 4.68) compared with other baseline models.

The remainder of this paper is organized as follows: Section 2 focuses on our main steps from image acquisition to model construction, and some details are used in the model training process; Section 3 lists the main results of our experiments; Section 4 discusses the potential limitations of our current work and future study; Section 5 summarizes our work.

## 2. Materials and Methods

### 2.1. Shrimp Fry Image Collection

**Figure 1:**
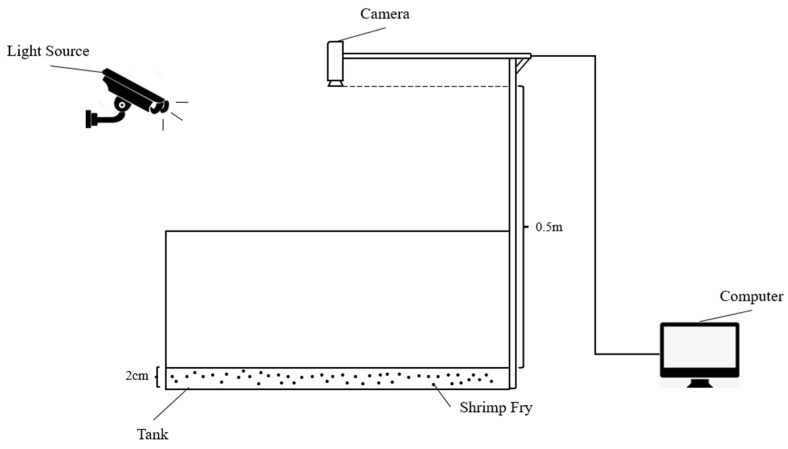
Experimental environment for acquiring images.

Shrimp fry images were collected from the Marine Biological Research Centre of Donghai Island, Zhanjiang City, Guangdong Province. As shown in Figure 1, the experimental environment for shrimp fry was set up indoors and mainly consisted of breeding tanks, cameras, LED light sources, and computers, which had the advantage of avoiding the effects of direct sunlight on the shrimp fry, as well as preventing the water from generating light spots that would affect the subsequent data processing. In order to obtain images under different shrimp density conditions, we adopted a strategy using an artificial method to increase or reduce the number of shrimp fry in the temporary breeding tanks. After shooting, the shrimp fry were returned to the original tanks immediately to avoid damage to them. The resolution of the camera was 720p, and it was located about half a meter above the water surface and remained vertical. In order to avoid the refraction of water that is too deep during the shooting process, we controlled the water depth to about 2 cm. The collected images were stored in jpg format; the images were collected during the day, and the shrimp fry were Penaeus vannamei. Under these environmental conditions, images that were not suitable for model training were eliminated. Finally, a total of 556 shrimp fry images were collected to form our dataset.

### 2.2. Image Dataset Annotation and Density Map Generation

In this section, we performed image annotation on the captured images. We used labelme software to label the location of the shrimp fry by point labeling, and the labeled files were stored in npy format. After the labeling was completed, we performed a statistical analysis of the images to determine the density of shrimp fry in each image collected. As shown in Table 1, we counted the range of shrimp fry numbers in each image and divided them into four different density levels: low, medium, high, and higher. It can be seen that images with different density levels appeared in the training set, validation set, and test set. This provided a better data basis for the subsequent training of the shrimp fry counting model and the testing of the accuracy of the model.

Inspired by the method proposed by Zhang [10], this paper used density maps generated for each shrimp fry image after annotation. Specifically, given the pixel position of the center of the shrimp fry in the image is $x_{i}$ and is represented by the function $\delta_{i}(x-x_{i})$, the density matrix of the jth image, which has N shrimp fry, is represented as follows:

(1) $$\begin{array}{c} H_{j}\left(x\right)=\sum_{i=1}^{N}\delta_{i}\left(x-x_{i}\right) \end{array}$$

After obtaining the density matrix $H_{j}\left(x\right)$ consisting of 0 and 1, the density matrix was subjected to Gaussian kernel blurring, and the Gaussian kernel function is denoted as follows:

(2) $$\begin{array}{c} G_{\sigma }\left(x_{i}\right)=\frac{1}{2\pi \sigma^{2}}e^{-\frac{x_{i}^{2}}{2\sigma^{2}}} \end{array}$$

The final density map-generating function can be expressed as follows:

(3) $$\begin{array}{c} F\left(x\right)=\sum_{i=1}^{N}\delta \left(x-x_{i}\right)*G_{\sigma }\left(x_{i}\right) \end{array}$$

**Figure 2:**
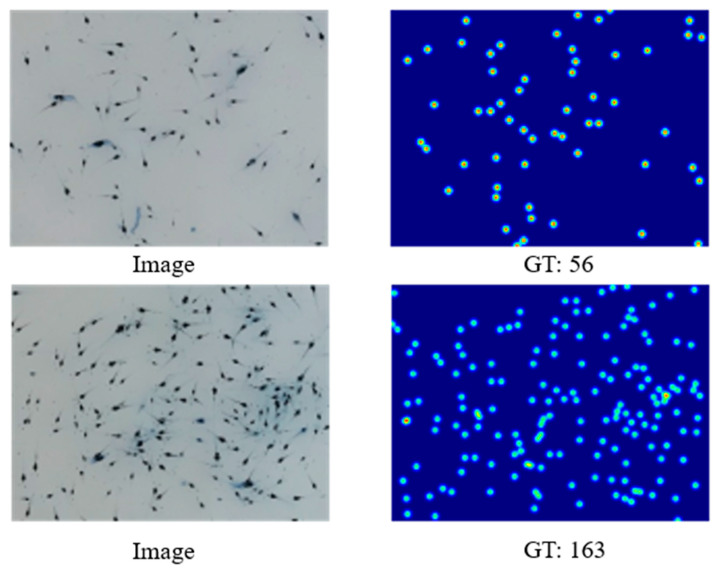
Shrimp fry images and their corresponding ground truth.

In this paper, we set the Gaussian kernel size to σ=3. To illustrate the relationship between our generated truth density maps and the original images, Figure 2 shows two images and their corresponding ground truth (GT) of density maps that we extracted from the dataset.

### 2.3. Shrimp Fry Counting Network

**Figure 3:**
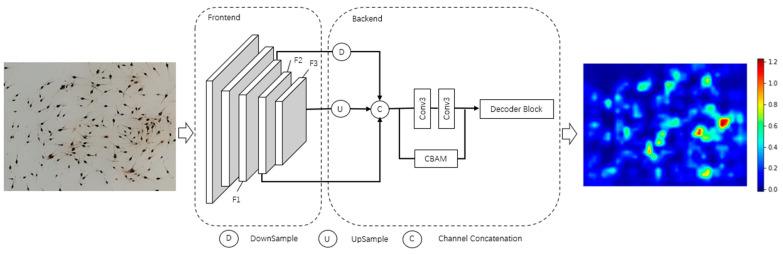
The architecture of the SFCNet.

This paper proposes a shrimp fry counting network (SFCNet) based on multi-scale attention fusion to solve the problems of low precision and low counting efficiency. The network was divided into two main parts: the frontend and the backend. The frontend network was used for feature extraction from the input shrimp fry images since Li [3] and Jiang [28] used VGG-16 [29] as a feature extraction network and achieved good results in the field of crowd counting. The first 13 layers of VGG-16 were used as the frontend network for feature extraction. The backend network extracted feature maps F1, F2, and F3 at different scales for fusion after the frontend network. The feature map output from F1 was down-sampled, and the feature map output from F3 was up-sampled and fused with F2 in the channel. Moreover, to pay more attention to the areas where shrimp fry are densely clustered and where there is occlusion between the shrimp fry, the CBAM attention module [30] was used to improve the counting accuracy of the dense regions. At the end of the backend network, we used five layers of inflated convolutional layers with an expansion rate of 2 to decode feature maps to the density map. The specific network structure is shown in Figure 3. The output is a density map of the same size as the original image. We also show the color bar in the image to reflect the distribution of shrimp fry at different densities in the image clearly.

### 2.4. Loss Function

The loss function used in this paper adds the structural similarity loss [31] ($L_{SSIM}$) to the Euclidean loss function ($L_{E}$) used to measure the degree of similarity between two images. In this paper, to measure the similarity between the ground truth of the density map and the predicted density map, the structural similarity loss function can be expressed as follows:

(4) $$\begin{array}{c} L_{SSIM}=1-\frac{1}{N}\sum_{x}SSIM \end{array}$$

where N is the number of training images, and the formula for SSIM is as follows:

(5) $$\begin{array}{c} SSIM=\frac{\left(2\mu_{p}\mu_{g}+c_{1}\right)\left(\sigma_{pg}+c_{2}\right)}{\left(\mu_{p}^{2}+\mu_{g}^{2}+c_{1}\right)\left(\sigma_{p}^{2}+\sigma_{g}^{2}+c_{2}\right)} \end{array}$$

where $\mu_{p}$ and $\sigma_{p}^{2}$ denote the mean and variance of the predicted density map, $\mu_{g}$ and $\sigma_{g}^{2}$ denote the mean and variance of the ground truth, $\sigma_{pg}$ denotes the covariance between the predicted density map and ground truth, and $c_{1}$ and $c_{2}$ are constants.

Thus, the final loss function can be expressed as follows:

(6) $$\begin{array}{c} L=L_{E}+\alpha L_{SSIM} \end{array}$$

Here, we set the hyperparameter α=0.001 to balance the orders of magnitude of the two different loss functions.

### 2.5. Evaluating Metrics

The evaluation metrics used in this paper are the mean absolute error (MAE) and root mean square error (RMSE). The MAE reflects the accuracy of the prediction error of the shrimp fry quantity, and the RMSE reflects the distribution of the prediction error. The formulas for the MAE and RMSE are as follows:

(7) $$\begin{array}{c} \mathrm{MAE}=\frac{1}{N}\sum_{i=1}^{N}\left|y_{i}-{\hat{y}}_{i}\right| \end{array}$$

(8) $$\begin{array}{c} \mathrm{RMSE}=\sqrt{\frac{1}{N}\sum_{i=1}^{N}{\left(y_{i}-{\hat{y}}_{i}\right)}^{2}} \end{array}$$

where $y_{i}$ and ${\hat{y}}_{i}$ denote the number of real shrimp fry and the predicted number of shrimp fry, respectively.

### 2.6. Procedure

**Figure 4:**
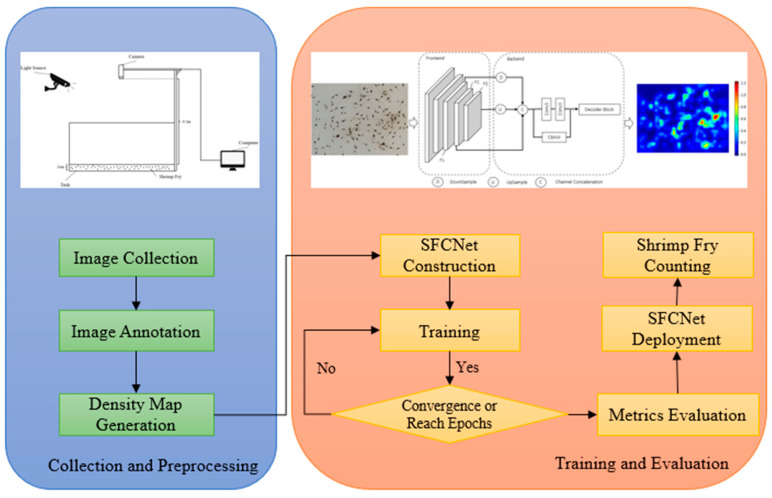
Main steps of image collection and annotation, and SFCNet training and evaluation.

In this section, we summarize the main steps from data acquisition to training our model and applying the model to actual shrimp fry counting. Figure 4 lists the main steps of our main operations. In the image collection and preprocessing stage, we used the image acquisition system constructed in Section 2.1 to collect the shrimp fry images, after which we labeled the locations of the collected shrimp fry images and then used the labeled files to generate the corresponding density maps of the shrimp fry images. In the model training and evaluation stage, we first constructed our SFCNet model, which was followed by model training. The training process was stopped when the model converged or reached the number of training epochs, and the model was evaluated with metrics to obtain the trained SFCNet.

## 3. Experiments and Results

### 3.1. Configuration

The training process of the shrimp fry counting network model proposed in this paper was performed on a graphics processing unit (GPU) server, and the configuration is shown in Table 2.

### 3.2. Hyperparameter Settings

For the training of the SFCNet, as shown in Table 3, the resolution of the initial collected images was 1280 × 720, which was too large for training. Therefore, we applied a center crop to every image to reduce the training time of the model. The cropped image aspect ratio was 4:3 (the actual image resolution as the input of the model was 768 × 576), and then we set the model to train for 300 epochs. The learning rate was set to $10^{-7}$, the batch size was 10, the momentum size was 0.95, and the weight decay was $5\times 10^{-4}$. In addition, the stochastic gradient descent (SGD) algorithm was chosen as our model’s optimizer. In addition, we initialized the frontend network in the SFCNet with pretrained weights from ImageNet 1000. For the convolutional layer in the backend network, we set a Gaussian kernel with a standard deviation of 0.01 and a bias of 0 for initialization. Moreover, the proposed SFCNet is an end-to-end structure which allows for easier counting of shrimp fry images.

In addition, in the training process, we performed data augmentation on the training dataset, such as mirroring the original data and flipping the shrimp fry images by 180 degrees with a 50% probability, which expanded the dataset and effectively improved the generalization performance of the model.

### 3.3. Comparison of Models

**Figure 5:**
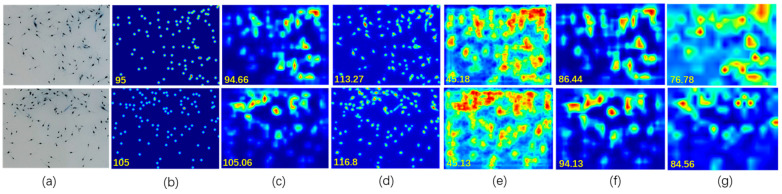
Comparison of density maps from different counting models. From left to right are (**a**) original images, (**b**) ground truth, (**c**) SFCNet, (d) U-Net, (**e**) MCNN, (**f**) CSRNet, and (**g**) VGG-16.

To illustrate the counting performance of our proposed model, we conducted a comparison with other baseline models to verify the ability of our model to count shrimp fry. Due to our regression-based counting method differing from the detection-based counting method in terms of evaluation metrics, we did not consider the detection-based counting method in our comparison scope. Table 4 lists the comparison results between the classical CNN models and our SFCNet; the evaluation metrics are defined in Section 2.5. From Table 4, it can be seen that although CSRNet achieves good results for crowd counting, for shrimp fry counting, the SFCNet achieves the best MAE and RMSE. The density map of the two shrimp fry images from the different models is shown in Figure 5, from which it can be seen that U-Net [24] is better for fitting ground truth than our model in terms of refinement. This is because our model is based on the original image through the maximum pooling to reduce the feature map to one-eighth of the original image and used the same method as Li [3] to directly resize the output feature maps to the size of the original image; however, our model had better counting performance compared with other models.

### 3.4. Ablation Study

In this section, we verified the effect of the modules in our SFCNet on the counting performance. We also conducted ablation experiments on the modules added to the SFCNet. Table 5 lists the results of the model after we used the multi-scale structure and added the CBAM attention module to the SFCNet. Clearly, the counting performance of the model was improved after we used the multi-scale structure and CBAM attention module. This was attributed to the fact that the different feature map output from the multi-scale structure could effectively address the aggregation of the shrimp fry, as well as the occlusion of the shrimp fry, while the attention module could make the model pay more attention to the above regions, thus improving the counting performance of the model and reducing the counting error.

**Figure 6:**
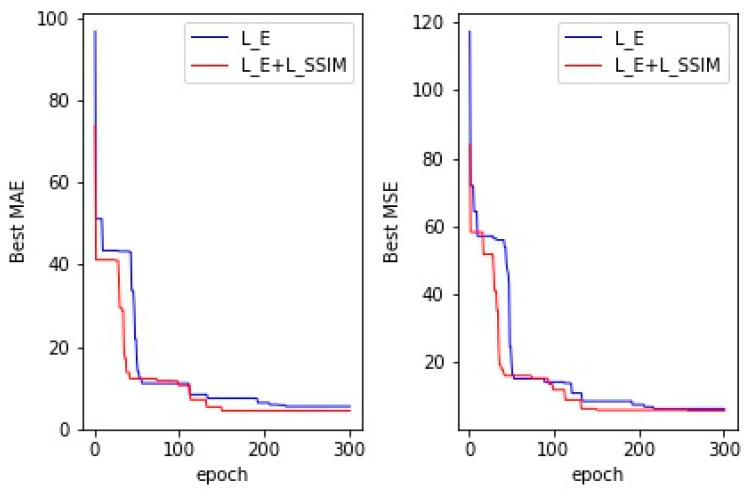
Convergence of the MAE and RMSE for the SFCNet using different loss functions.

In addition, to improve the convergence speed of our proposed model, we also conducted ablation experiments on the loss functions; Table 6 shows the loss functions used in our training of the SFCNet. We compared the results of training the model using the Euclidean loss function with the results of using both the SSIM loss and the Euclidean loss functions. Figure 6 shows the MAE and RMSE of the validation set of the SFCNet converged with the use of different loss functions. As shown in Table 6 and Figure 6, the SFCNet using Euclidean loss as well as SSIM loss not only obtained a better counting performance, because the structural similarity loss pays more attention to the finer-grained counting errors in the image, but also converged faster; thus, it was able to find the global optimal solution.

## 4. Discussion

Compared with the time-consuming and labor-intensive manual counting, the shrimp fry counting network constructed by a deep learning method provides a more effective method for evaluating shrimp fry growth status, adult shrimp yield estimation, and transportation management in aquaculture. Most of the previous studies on counting tasks are detection-based methods. The limitations of these are that the size of the counting objects is too small, or the shrimp fry are blocked from each other, which results in missing detection, or other objects are similar to the counting objects, which causes false detection. The regression-based counting method can effectively deal with this problem by modeling the images globally and integrating the final output density map to obtain the counting quantity with shrimp fry that are occluded by each other or shrimp fry that are too small. In order to objectively analyze our model, we also discuss the limitations of the current work and future studies.

### 4.1. Potential Limitations of Current Work

Although our SFCNet has a lower counting error than other traditional CNN models, the method proposed in this paper has the following three points that need to be improved: (1) our method is aimed at the stage of shrimp fry and cannot solve the problem of counting shrimp bodies in different environments and in different breeding periods; (2) compared with the traditional CNN model, the computational resources and inference time of the SFCNet are slightly increased, but the amount of increase is controllable and acceptable in practical applications. For example, the model computing resource of CSRNet on the shrimp fry counting is 12.7 MB, and the average inference time for the test set is 46 ms, while the model of the SFCNet is 44.1 MB and 90 ms; (3) since the number of shrimp fry in the dataset we constructed was mainly distributed in the hundreds, the SFCNet model could show good counting accuracy in the scenario with low density. However, we are aware that in the actual production environment, the density of shrimp fry can vary greatly, especially in high-density farming environments.

### 4.2. Future Study

In view of the limitations of the analysis in Section 4.1, our future studies will focus on the following aspects: (1) considering the quantity monitoring of different species of shrimp fry in different water breeding environments, the model will be extended to different species and environments to enhance the robustness and applicability; (2) we will continue exploring methods to reduce the computing resources and the time of the model while improving the counting performance of the model, such as network structure and hyperparameter settings, to make the model more lightweight; (3) in order to evaluate the counting performance of the SFCNet in high-density shrimp fry scenarios comprehensively, we plan to add more high-density shrimp fry image data in future studies and optimize and adjust the model accordingly. By expanding the scope and diversity of the dataset, we can accurately simulate the complexity of the actual farming environment, allowing for a more comprehensive assessment of the model’s generalization ability and counting accuracy.

## 5. Conclusions

In this paper, we proposed a multi-scale attention fusion method for shrimp fry counting. The network consisted of a frontend and a backend. We chose VGG-16 as our backbone in the frontend, and in the backend, we added a multi-scale structure and a CBAM module and used five layers of the dilated convolutional layer to generate our final density map. Then, we evaluated the proposed network with our self-constructed shrimp fry dataset. Through experiments and analysis, we could see that our proposed network (SFCNet) was able to count the number of shrimp fry in a filmed scene better, with fewer counting errors than other baseline networks. We also conducted ablation experiments on the multi-scale structure, as well as the CBAM attention module used in our proposed network, and compared the speed of model training and the counting error using different loss functions; the results showed faster convergence and a smaller counting error in the case of model training with the use of the joint Euclidean loss as well as the SSIM loss. Therefore, our proposed model can better meet the demand for shrimp fry counting in a real production environment.

## Figures and Tables

**Table 1:** Statistics of density levels of shrimp fry images and the division of training set, validation set, and test set.

Density Level	Range	Number of Images		
		Train	Validation	Test
Low	[0, 249]	121	21	55
Medium	[250, 499]	139	15	11
High	[500, 749]	105	14	19
Higher	[750, 1000]	25	13	18

**Table 2:** The configuration of hardware for model training.

Configuration	Type
OS	Ubuntu 20.04
CPU	Intel(R) Xeon(R) Gold 6338
GPU	NVIDIA A30 24G
Memory	40G
Platform (computing)	Pytorch 1.8
Programming Language	Python 3.9

**Table 3:** Hyperparameter settings for training the SFCNet.

Parameter	Value
input size	768 × 576
epoch	300
batch Size	10
learning rate	$10^{-7}$
momentum	0.95
optimizer	SGD
weight decay	$5\times 10^{-4}$

**Table 4:** Comparison of different models on the shrimp fry dataset.

Model	MAE	RMSE
MCNN [32]	20.859	25.675
U-Net [24]	6.746	9.068
VGG-16 [29]	5.966	7.204
CSRNet [3]	5.704	7.358
SFCNet (ours)	**3.960**	**4.682**

**Table 5:** Effects of adding different modules to the SFCNet on counting performance.

Frontend	Backend	MAE	RMSE
VGG-16	—	5.966	7.204
VGG-16	Multi-scale	5.167	6.644
VGG-16	CBAM	5.016	6.84
VGG-16	Multi-Scale + CBAM	**3.96**	**4.682**

**Table 6:** MAEs and RMSEs of the SFCNet using different loss functions.

Model	Loss	MAE	RMSE
SFCNet	$L_{E}$	4.372	5.846
	$L_{E}+L_{SSIM}$	3.96	4.682

## Data Availability

The data that support the findings of this work are available upon request.
